# Maternal Long-Chain Polyunsaturated Fatty Acids Status in Pregnancy and Newborn Body Composition

**DOI:** 10.3390/nu17010066

**Published:** 2024-12-27

**Authors:** Mira Dewi, Nuri Andarwulan, Utami Wahyuningsih, Renata Kazimierczak, Dominika Średnicka-Tober

**Affiliations:** 1 Faculty of Medicine, IPB University, IPB Dramaga Campus, Bogor 16680, West Java, Indonesia; mirade@apps.ipb.ac.id; 2 Southeast Asian Food and Agricultural Science and Technology (SEAFAST) Center, IPB University, IPB Dramaga Campus, Bogor 16680, West Java, Indonesia; 3 Department of Food Science and Technology, Faculty of Agricultural Technology, IPB University, IPB Dramaga Campus, Bogor 16680, West Java, Indonesia; 4 Faculty of Health Science, Universitas Pembangunan Nasional Veteran Jakarta, Jakarta 12450, West Java, Indonesia; utamiwahyuningsih@upnvj.ac.id; 5 Department of Functional and Organic Food, Institute of Human Nutrition Sciences, Warsaw University of Life Sciences, Nowoursynowska 159c, 02-776 Warsaw, Poland; renata_kazimierczak@sggw.edu.pl

**Keywords:** diet, LC-PUFA, newborn, body composition, pregnancy, Indonesia

## Abstract

Background: A number of clinical studies have shown a positive association between the maternal *n*-3 PUFA status during pregnancy and fetal and newborn development and health. Despite this well-documented role of *n*-3 PUFAs in pregnancy, data on maternal the LC-PUFAs status during pregnancy in the Indonesian population, to our knowledge, are not yet available. This study reports on the LC-PUFA dietary intake among pregnant women in a suburban population of Bogor City, West Java, Indonesia. It also explores the associations of maternal LC-PUFA intake with maternal blood, cord blood, and breast milk LC-PUFA levels and the associations of the latter with infant body composition. Methods: A total of 142 pregnant women and 104 newborn infants were included in this study. The dietary intake of energy, macronutrients, and selected LC-PUFAs (LA, ALA, EPA, and DHA) was assessed by 2 × 24 h food recall and FFQ. LC-PUFA levels were measured in maternal blood, cord blood, and breast milk. Newborn body composition was determined by anthropometric measures. Results: The study found that the inadequate intake of energy, protein, and carbohydrates was highly prevalent among pregnant women subjects. The intake of the most important *n*-3 PUFAs (ALA, EPA, and DHA) was far below the recommended values. Maternal dietary DHA intakes were negatively associated with birth weight and infant fat mass at birth, and dietary intake of total fat and *n*-6 LA were associated with increased fat accumulation in newborns at specific body sites. Moreover, positive correlations were identified between the EPA in maternal blood and infant % fat mass, and between the DHA in cord blood and newborn birth weight. Conclusions: Further longitudinal studies, including clinical and biomolecular analyses, are suggested to be conducted to monitor maternal and child health and nutrition in Indonesia and develop well-attuned intervention strategies.

## 1. Introduction

Long-chain polyunsaturated fatty acids (LC-PUFAs), belonging to both the *n*-3 (ω-3) and the *n*-6 (ω-3) families, are known to have an essential role in fetal growth and development [1,2,3,4]. During the last trimester of pregnancy, docosahexaenoic acid (DHA), representing the *n*-3 class of LC-PUFAs, accumulates in parts of the fetal brain responsible for attention, working memory, and inhibitory control [5]. It has been shown to stimulate cellular processes important for proper neuronal development and function [4], thus further impacting children’s intellectual performance [6]. A number of studies have shown a positive association between maternal *n*-3 PUFA status during pregnancy and reduced risks of preterm births, perinatal death, neonatal care admission, and low birth weight [7,8]. Maternal *n*-3 PUFA intake was also shown to prevent offspring’s adiposity by modulating the glucose and lipid metabolism, but also by impacting the fetal development of thermogenesis and skeletal growth dynamics [9].

Despite the well-documented role of the maternal status of *n*-3 PUFAs during pregnancy for offspring development and health, data on maternal LC-PUFAs intake during pregnancy in the Indonesian population, to our knowledge, are not yet available. Indonesia is one of the largest archipelagic states in the world. However, the consumption of fish, the main dietary source of *n*-3 PUFAs, in Indonesia is relatively low (41.11 kg/cap/year) compared to its neighboring countries (70 kg/cap/year in Malaysia and 140 kg/cap/year in Japan) [10]. In a study conducted in 2005, it was found that PUFA intake in Indonesia was lower than in other Asian countries, reaching 5.35 g/d [11]. A recent study conducted in Jakarta, the capital of Indonesia, found that the intake level has not changed significantly, being estimated as 5.75 g/d [12]. Based on these data, we assume that the maternal dietary intake of LC-PUFAs is also low.

In addition to its effect on offspring development, there is growing evidence that the maternal dietary fatty acid composition also determines the offspring’s body composition [13]. The increased intake of *n*-6 PUFA relative to *n*-3 PUFA, as seen in modern diets, promotes fat cell differentiation and accumulation [14]. Based on this evidence, increasing *n*-3 intake during pregnancy has been proposed as a strategy to promote a favorable offspring body composition and reduce the problem of childhood obesity. A number of *n*-3 intervention studies during pregnancy and offspring body composition have been conducted [15]. However, the body composition in those studies was measured in childhood when the influence of the extra-uterine environment, such as feeding practice, has already interfered. To investigate the role of the intra-uterine environment, it is important to measure the outcome soon after birth, i.e., in the newborn.

We aim to provide data on maternal LC-PUFA intake during pregnancy and pregnancy/birth outcomes. The rationale of this study is that understanding the maternal nutrition status and newborn body composition association will facilitate the development of effective dietary strategies specific to Indonesian mothers.

## 2. Materials and Methods

### 2.1. Study Design and Subjects

This was an observational study of a total of 142 women in the Bogor district, Indonesia. Women were recruited at their third pregnancy trimester and were followed until they gave birth. Women who came for antenatal care to two public health services (Puskesmas), i.e., Bogor Utara and Tanah Sereal, were invited to participate in the study if they were 18–40 years old and with a singleton pregnancy. Women were excluded if they had health conditions that may have influenced the growth and development of the fetus, such as undernutrition (a body mass index below 18), diabetes mellitus, congenital abnormalities, or cancer before or during pregnancy. The study was approved by the Human Research Ethics Committee of IPB University, Bogor, Indonesia (protocol code No. #041/IT3/KEPMSM-IPB/SK/2018 issued on 3 April 2018).

During the study, subjects were scheduled for three visits: visit 1 conducted in Puskesmas during their third trimester of pregnancy, for an interview and peripheral blood sample collection; visit 2 conducted in Puskesmas/clinics/hospitals at birth, for cord blood sample collection; and visit 3 conducted by home visit 5–14 days after birth, for breast milk sample collection and newborn anthropometric assessment.

**Figure 1:**
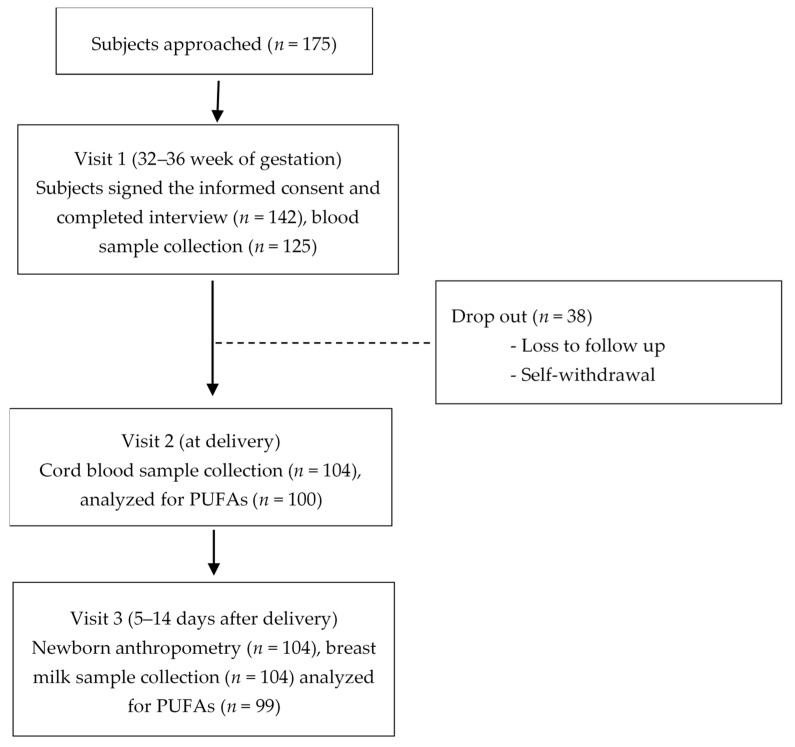
Study design diagram.

Of the 175 eligible women approached, 142 provided their consent and completed visit 1 for dietary intake, but only 125 showed up for blood sample collection. Later, 38 women dropped out due to loss to follow-up and self-withdrawal. At the birth visit, cord blood samples from 104 women were collected; however, four samples were not analyzed due to hemolysis. At the final visit, 104 mothers and their newborn infants were visited, but only 99 breast milk samples were collected due to a lack of lactation in the case of five mothers (Figure 1).

### 2.2. Dietary Intake of LC-PUFAs, Energy, and Macronutrients

Data on the maternal dietary intake of LC-PUFAs were collected using a 2 × 24 h food recall and food frequency questionnaire (FFQ) at 32–36 weeks of gestation. The intake levels of energy, carbohydrates, protein, fat, and LC-PUFAs such as linoleic acid (LA), α-linolenic acid (ALA), eicosapentaenoic acid (EPA), and docosahexaenoic acid (DHA) were calculated based on the Indonesian Food Composition Table 2017 [16], Indonesian Fatty Acids Composition Table [17], and Australian Food Composition Database AUSNUT 2011 [18], and were analyzed using NutriClin3 version 1.0 software.

### 2.3. Sampling and Biomarker Analysis

#### 2.3.1. Maternal Blood Sample Collection

Between 32 and 36 weeks of gestation, approximately 3 mL of blood sample was drawn from every subject’s cubital vein. The blood samples were collected in labeled EDTA tubes to prevent coagulation and immediately centrifuged to separate blood cells from plasma. After the removal of the buffy coat, the remaining red blood cells were placed in cups, labeled, and stored in a refrigerator for a maximum of 6 h before being transported to a −20 °C freezer, where the samples were kept for a maximum of one week until analysis.

#### 2.3.2. Cord Blood Sample Collection

About 3 mL of cord blood was collected from each subject within 10 min after delivery, from the placental portion of the umbilical cord, by venipuncture, immediately after clamping. The blood samples then underwent the same process as the maternal blood samples.

#### 2.3.3. Breastmilk Sample Collection

The breast milk samples were collected during the final visit of the subjects on days 5–14 after delivery (visit 3). Subjects were asked to express 3–5 mL of breast milk. The breast milk was collected into labeled vial tubes and then stored in a refrigerator for a maximum of 6 h before being transported to a −20 °C freezer, where the samples were kept until analysis.

#### 2.3.4. LC-PUFAs Analysis

The extraction of lipids from the red blood cell and breastmilk samples was performed using the modified Folch method [19]. The samples were methylated with MeOH-HCL 6N, and then extracted using chloroform–methanol solvent. The supernatant containing fatty acid methyl esters (FAMEs) was evaporated under nitrogen gas and redissolved in *n*-heptane to be injected into the gas chromatography flame ionization detector (GC-FID) machine. After the analysis, the peak point of the FAMEs was identified by comparing the retention time according to the certified standards. The fatty acid percentage was calculated.

### 2.4. Birth Outcomes

Data on birth outcomes, such as birth weight, length, and head circumference, were obtained from medical records. At the final visit (5–14 days postpartum), anthropometric assessments such as body weight, body length, and skin fold thickness (triceps, subscapular, thigh) were performed. Infant body weight and length were measured using an infant digital scale and an infant length board. All measurements were performed twice, and the average values were taken for analysis. A difference between the two measurements of up to 0.5 cm was accepted. Body fat was estimated by skin fold thickness (triceps, subscapular, and thigh) assessment using a Lange^®^ body caliper, performed by trained staff. The body fat mass was calculated using the Aris et al. [20] equation as follows:

−0.022 + 0.307 × weight (kg) − 0.077 × gender (1 = male; 0 = female) − 0.019 × gestational age (week) + 0.028 × subscapular skin fold (mm)

### 2.5. Statistical Analyses

Data were analyzed using Microsoft Excel and SPSS version 16.0 software (SPSS Inc., Chicago, IL, USA). Data on subject characteristics, the dietary intake of LC-PUFAs, the level of LC-PUFAs, and birth outcomes are presented descriptively. The levels of LC-PUFAs in maternal blood, cord blood, and breast milk were compared with one-way ANOVA followed by Tukey’s HSD test. The correlations between levels of LC-PUFAs in maternal blood, cord blood, and breast milk, as well as between dietary intake, blood LC-PUFAs, and newborn outcomes, were assessed by Pearson or Spearman correlation, depending on the data distribution tested by Kolmogorov–Smirnov normality analysis. For all tests, two-tailed *p*-values ≤ 0.05 were considered statistically significant.

## 3. Results

### 3.1. Subjects Characteristics

The characteristics of 142 pregnant women subjects who came for the first study visit in their third trimester are presented in Table 1. The women were 29.4 ± 6.2 years old on average, primarily housewives (79.6%), and the majority had a family income below the minimum regional standard (77.5%). Most finished their education at senior high school or lower levels, while only 8.5% finished college. Almost all women were non-smokers (97.2%). Before their current pregnancy, 24.6% of women were overweight, and 6.3% were obese.

Due to various reasons, 38 women dropped out before delivery, and thus only 104 women and their newborn infants were visited at delivery. All infants were born alive and without apparent congenital disorders. About half were males. Fifteen infants (14.4%) were born by C-section, while the rest through vaginal delivery. One infant was born prematurely (36 weeks) but had a normal birth weight (2700 g). Also, there was one infant whose birth weight was 2450 g, i.e., just below the cut-off of normal weight (2500 g). About one-fifth of infants were born short (<48 cm).

Postpartum visits were conducted between days 5 and 14 after birth (6.2 ± 1.4 days on average). Infants’ anthropometric parameters measured during the visits are shown in Table 2. As expected, the infants’ weight and length were higher on average than those measured at birth. Skin fold thickness was measured from three sites, i.e., the triceps, subscapular, and thigh. On average, the thigh skin fold was thicker than the other two sites, indicating that more fat is accumulated in the thigh than in the triceps and subscapular. Infant fat mass was calculated using a formula by Aris et al. [20]. According to the formula, the infant fat mass is determined by the body weight, gender (females have, on average, more fat than males), gestational age at birth, and subscapular skin fold thickness. The calculated fat mass percentage of the infants ranged between 1.3% and 20.3%, with an average of 10.7 ± 3.7%. Our previous study and others showed that the anthropometric fat mass estimation, although considered valid, may be imprecise, particularly for low fat mass (FM) values. The one subject whose %FM was estimated as 1.3% (extremely low) was a male, who had a body mass of 2650 g at 41 weeks of gestational age, and had a thigh skin fold of 3.5 cm. While the measurements were within an acceptable range, the resulting very low value (1.3% FM) is likely imprecise [21].

### 3.2. Maternal Dietary Energy, Macronutrients and PUFAs Intake

Data on maternal energy, macronutrients, and LC-PUFA intake are presented in Table 3. The subjects’ intake of energy and macronutrients was, on average, below the recommendations (RDI), except for fat, which was nearly the same as the RDI.

In general, most women did not meet an adequate intake for energy and macronutrients. More than 60% of women had an inadequate intake of energy. Only 14.1% of women had an adequate intake of carbohydrates, and 35.9% had an adequate protein intake. On the other hand, over 70% reached an adequate intake of fat.

The subjects’ median intake of LA exceeded the recommendation, i.e., 10.4 g/d (RDI = 8.5 g/d). Over 60% of women met daily recommendations for LA intake. On the other hand, less than 5% of women achieved an adequate intake for ALA, EPA, and DHA. The women’s median intake of ALA was less than half of the recommendation, i.e., 0.6 g/d while the recommendation is 1.4 g/d. Furthermore, the subjects’ median intake of *n*-3 PUFAs was way lower than the recommendation, i.e., 2.1 mg/d for EPA and 38.2 mg/d for DHA, while the recommendations were 100 mg/d and 200 mg/d for EPA and DHA, respectively (Table 3).

### 3.3. Maternal Blood, Cord Blood, and Breast Milk Levels of PUFAs

**Figure 2:**
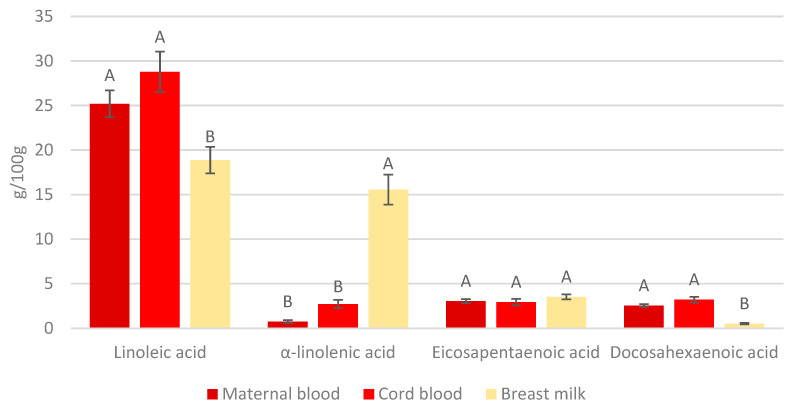
Levels of LC-PUFAs in maternal blood (*n* = 125), cord blood (*n* = 100), and breast milk (*n* = 99) (g/100 g); mean ± SE. Different letters above bars representing the same fatty acid show statistically significant differences (Tukey’s HSD test, *p* < 0.05).

We analyzed LC-PUFA levels in maternal blood samples collected during their third trimester of pregnancy, in cord blood samples collected right after delivery, and in breast milk samples. Altogether, 125, 100, and 99 samples of maternal blood, cord blood, and breast milk, respectively, were included in the biomarker analysis. The results are presented in Figure 2.

Among the four analyzed LC-PUFAs, LA presented the highest levels in all types of samples. The level of LA in maternal blood during pregnancy was not statistically different compared to that in cord blood, while its level in breast milk was significantly lower. On the contrary, the average level of ALA in the cord blood was not different from that in maternal blood during pregnancy, while in breast milk, its level was significantly higher. No significant differences were found among the levels of EPA in the three types of samples. The average level of DHA in breast milk was significantly lower than in maternal blood and cord blood, while no difference was found between DHA levels in maternal blood and cord blood.

Among the four fatty acids analyzed in maternal blood samples, LA was found at the highest levels, while ALA presented the lowest concentrations. Similarly, the LC-PUFAs found at the highest and the lowest levels in cord blood were LA and ALA, respectively. On the other hand, breast milk had comparable levels of ALA and LA, but lower DHA and EPA levels.

No significant correlations were found between LA, ALA, EPA, and DHA levels in maternal blood with those in cord blood and breast milk (Table 4).

### 3.4. Associations of Maternal Dietary Intake of LC-PUFAs with Levels of LC-PUFAs in Maternal and Cord Blood

The Spearman correlation test was used to determine the associations between maternal dietary LC-PUFA intake and maternal and cord blood levels of LC-PUFAs (Table 5). We found no significant associations between the dietary intake and maternal blood levels of LC-PUFAs. At the same time, the dietary LA and ALA intakes were positively associated with their levels in cord blood. On the contrary, the dietary DHA intake was negatively associated with its cord blood level.

### 3.5. Associations of Maternal Dietary Intake, Maternal LC-PUFAs Levels, and Cord Blood LC-PUFAs Levels with Newborn Body Composition

No significant correlation was found between maternal dietary energy, protein, fat, and carbohydrate intake during the third trimester of pregnancy and the infant fat mass percentage (Table 6). Maternal dietary fat intake, however, was associated with a higher infant subscapular skin fold thickness (SFT), which indicated a higher fat content in this body area (r = 0.194; *p* = 0.048).

The maternal dietary intake of linoleic acid was positively associated with the infant triceps SFT, subscapular SFT, and thigh SFT (r = 0.203, 0.203, and 0.254, respectively; *p* < 0.05) and the intake of α-linolenic acid was positively associated with thigh SFT (r = 0.213, *p* = 0.030), while the intake of DHA was negatively associated with the infant birth weight (r = −0.221, *p* = 0.024). There was also a trend toward a negative association between the maternal intake of DHA and the infant % fat mass (r = −0.192, *p* = 0.051).

A correlation analysis of maternal blood LC-PUFAs and infant anthropometrics and body composition is presented in Table 7. Maternal blood EPA levels were positively correlated with infant thigh skin fold thickness (r = 0.210, *p* = 0.034) and infant percentage of fat mass (r = 0.247, *p* = 0.012).

Table 8 shows no significant associations between the cord blood LC-PUFA levels and most infant anthropometrics and body composition parameters. In an exception, the DHA levels in cord blood were positively correlated with the birth weight (r = 0.272, *p* = 0.006).

## 4. Discussion

This study reports on the LC-PUFA dietary intake among pregnant women in a suburban population of Bogor City, West Java, Indonesia. It also explores the associations of maternal LC-PUFA intake with maternal blood, cord blood, and breast milk LC-PUFA levels and the associations of the latter with infant body composition at the age of 5–14 days.

An adequate intake of energy and nutrients holds an important role during pregnancy. It is essential that women meet their dietary requirements during this critical period, or else it may compromise both maternal and fetal health outcomes. The present study showed that an inadequate intake of energy, protein, and carbohydrates was highly prevalent among the study subjects. This might have been caused by the fact that most of the women involved in this study had family incomes below the minimum regional standard. Family income drives households’ purchasing power, which in turn affects the quality and quantity of the food consumed. This is in line with the report by the National Statistics Agency, which suggested that the lowest per capita consumption of energy and protein was found among people with the lowest quintile of expenditures [24].

Most of the women in the study had adequate total fat intake. However, improvement was identified as needed in terms of *n*-3 PUFA intake. The intake of ALA, EPA, and DHA, three of the most important *n*-3 PUFAs, was far below the recommended levels. EPA and DHA can be found in animal-based foods, mainly fish and other seafood, which are generally more expensive than plant-based foods. Hence, the low intake of these fatty acids might also have been partly related to the low purchasing power among the study participants. According to the National Statistics Agency report, the low consumption of animal-based food such as fish, other seafood, meat, eggs, and milk was also associated with low income levels [24].

Studies on maternal PUFA intake among the Indonesian population are still limited. An observational study on maternal PUFA intake among pregnant women in Jakarta pointed to the intakes of total *n*-3, ALA, EPA, and DHA being far below the recommended daily levels [25], which stays in line with the results presented here. At the same time, Forsyth et al. [26] estimated the daily intake of DHA in the Indonesian population at a level of 240.8 mg/day, which falls within the recommended range of 200–250 mg/day. However, this was an estimate for the whole country, not taking into consideration the situation of specific population groups and within-country regions.

On the other hand, although many studies have concluded about the benefits of *n*-3 PUFA during pregnancy, the awareness of the importance of *n*-3 PUFA intake among pregnant women is still low. Previous studies suggested that the intake of *n*-3 PUFA could reduce the prevalence of preterm birth, perinatal death, neonatal care admission, and low birth weight [7]. Maternal *n*-3 PUFA intake was also shown to prevent offspring’s adiposity by modulating the glucose and lipid metabolism, but also by impacting the fetal development of thermogenesis and skeletal growth dynamics [9].

A low intake of *n*-3 PUFA, resulting in an increased *n*-6/*n*-3 ratio, was found to promote the pathogenesis of several diseases. The *n*-6 fatty acids are known to have pro-inflammatory properties, as opposed to *n*-3 fatty acids, which show anti-inflammatory potential [27,28]. Diets with high levels of *n*-6 PUFA before and/or during pregnancy have been suggested to have detrimental effects on fetal development and programming that may influence the health of offspring in adulthood [28,29].

The maternal blood LC-PUFA levels reported in this study differ from those reported in other studies performed in Indonesia. However, these studies included pregnant women at different stages of pregnancy (first trimester). Moreover, they neither clearly described the type of sample being analyzed (plasma or erythrocyte) nor the method of analysis [30].

In comparison to the results of a study involving Japanese pregnant women, the level of maternal and cord blood *n*-6 fatty acids, i.e., linoleic acid, in our study population was much higher (8.95% vs. 25.2% on average for the maternal blood level and 4.05% vs. 28.79% on average for the cord blood level), while the level of DHA both in maternal and cord blood, on the contrary, was lower (6.31% vs. 2.55% on average for the maternal blood level and 6.92% vs. 3.20% on average for the cord blood level) [31]. Interestingly, the maternal levels of EPA in comparison to DHA in this study, as well as in the other two studies involving the Indonesian population, were found to be higher, while most other studies reported that maternal blood EPA levels were consistently lower than DHA levels. The different findings might be related to genetic variations among races and need to be further investigated through molecular studies of LC-PUFA metabolism and synthesis, specifically in the Indonesian population [30,31,32,33].

The cord blood level of PUFA was meant to represent the PUFA level entering the infant’s body. Cord blood acts as the vehicle that carries PUFA and other nutrients from the maternal to the infant body throughout the course of pregnancy. Correlations of LC-PUFAs in maternal blood and cord blood were reported in previous studies; however, we did not observe such correlations in our subjects [31]. The fetal blood fatty acid composition is highly determined by the supply from the mother. However, other aspects also contribute to each specific PUFA uptake and distribution. These factors include metabolic conversion, incorporation, and oxidation, which vary among different genotypes, especially for FADS [34,35]. The consequence of genotype variations among pregnant women in the Indonesian population is still understudied and, therefore, needs further investigation.

The distribution of LA, ALA, EPA, and DHA in maternal erythrocytes was similar to that in the cord blood but was different in breast milk. In comparison to maternal blood and cord blood, the levels of *n*-6 LA and *n*-3 DHA were lower in breast milk; however, the level of ALA, which is the precursor for EPA and DHA, was much higher. This finding indicates a unique metabolic pathway of PUFA in breast milk, which is different from that in maternal blood and cord blood. During the exclusive breastfeeding period, breastmilk is the sole source of nutrients for infants. Breastmilk PUFA content is expected to be high enough to fulfill the infant’s requirements in this period.

The maternal intake of dietary fatty acids during pregnancy has been reported to modulate their levels in maternal blood, and their supply to the fetus [36]. We observed that the maternal dietary PUFA intake has more influence on the PUFA level in cord blood than in maternal blood. LA and ALA were positively correlated with the levels in cord blood. However, unexpectedly, dietary DHA was found to be negatively correlated with its level in the cord blood. This result needs to be further analyzed by looking at other factors (e.g., dietary patterns, especially other fatty acids, lifestyle, concurrent diseases, metabolic conditions) that could also have impacted both variables in each individual.

Our main findings regarding maternal dietary LC-PUFAs and the newborn body composition relationship are that dietary DHA was negatively associated with the infant fat mass at birth. We also found that a higher dietary intake of total fat and *n*-6 LA was associated with higher infant fat at specific body sites. The findings support the previous findings from animal studies that the increased intake of *n*-6 PUFA relative to *n*-3 PUFA promoted fat cell differentiation and accumulation [14].

The EPA level in maternal blood was associated with higher infant fat mass, but EPA in cord blood was not. Also, maternal EPA was not associated with cord blood EPA, indicating that the fatty acids underwent a metabolic process when transferred from the maternal to the fetal compartment. Furthermore, no significant association was found between PUFA levels in the cord blood and the infant fat mass. However, the DHA level was associated with a higher birthweight but not fat mass, indicating that the increase in weight was that of fat-free mass and not fat. We did not analyze the association between LC-PUFAs in breast milk and newborn fat mass, as the breast milk sample was collected at a relatively short period of time after birth, and thus its effect on infant adiposity may not yet be clinically observable.

Our study provides scientific evidence that the increase of *n*-3 fatty acids intake during pregnancy is associated with a favorable offspring body composition that, in the long term, could possibly help to reduce the problem of childhood obesity. Since this study measured body composition in newborns when the influence of the extra-uterine environment, such as feeding practice, has not yet been significant, the findings highlight the influence of the intra-uterine environment on the outcomes. However, we could not demonstrate clear evidence that the LC-PUFA status in the maternal and fetal compartments predicts infant adiposity. Like most other metabolic compounds in the body, the levels of maternal blood PUFAs were suggested to change throughout the pregnancy. Since we only took a one-time measurement, i.e., in the third trimester, we were not able to obtain a more comprehensive description of maternal PUFA level dynamics and determine if there is any specific time point where the fatty acids play a role in fat deposition in the developing fetus.

There are some other limitations that are important to consider. First of all, we only included four fatty acids in the analysis, i.e., LA, ALA, EPA, and DHA, while there are many other fatty acids and fatty acid indexes that also play important roles in the maternal and fetal metabolism, particularly in fat metabolism and deposition. In addition, the determination of fatty acids in the diet was based on food conversion data from different countries, which may not be accurate when applied to Indonesian food. In addition, this study followed the subjects for only a relatively short period of time, and hence, the longer-term effect of maternal PUFA status could not be demonstrated.

Maternal and child health and nutrition are still among the main concerns in Indonesia. The problems of stunting and anemia in pregnancy and infants are still among the priorities to be solved, while the problem of child obesity is increasing at the same time. Our study provides important information that helps increase our understanding of the maternal status of LC-PUFAs, specifically in a group of women in the Indonesian population, both from diet and biochemical indicators, and how these factors are associated with pregnancy/birth outcomes. Pregnancy is a critical period for human development, and the fulfillment of nutrition needs during this period is important in order to obtain optimal health for both the mother and the developing fetus. We suggest that further longitudinal studies, including clinical and biomolecular analyses, should be conducted in the near future to help improve maternal and child health and nutrition.

## 5. Conclusions

The study has shown a high prevalence of an inadequate intake of energy, protein, and carbohydrates among pregnant women subjects. The intake of the most important *n*-3 PUFAs (ALA, EPA, and DHA) was also far below the recommended values.

No consistent associations were observed among the levels of the four PUFAs (LA, ALA, EPA, DHA) in the maternal diet vs. maternal blood during pregnancy, cord blood, and breast milk.

Higher maternal dietary DHA intakes were associated with a lower infant fat mass at birth, and a higher dietary intake of total fat and *n*-6 LA was associated with higher infant fat at specific body sites. Thus, the increase in *n*-3 fatty acids intake during pregnancy is suggested to be associated with a favorable offspring body composition.

Further longitudinal studies, including clinical and biomolecular analysis, and taking into consideration specific genotypes of Indonesian women, are suggested to be conducted in the near future to help improve maternal and child health and nutrition, and develop well-attuned intervention strategies.

## Figures and Tables

**Table 1:** Study subjects’ characteristics.

Characteristics	*n*	%
Mothers (*n* = 142)		
Age (years), mean ± SD	29.4 ± 6.2	
Education		
Elementary school	38	26.8
Junior high school	24	16.9
Senior high school	68	47.9
Diploma/college	12	8.5
Working status		
Housewife	113	79.6
Employee	29	20.4
Family income below minimum regional income (<3,599,400 IDR)	110	77.5
Smoking habit	4	2.8
Pregnancy history		
Parity ≥ 1	97	68.3
History of Abortion	17	17.2
Gestational age at visit 1 (week), mean ± SD	37.0 ± 2.0	
Pre-pregnancy BMI (kg/m^2^)	26.9 ± 2.9	
Normal (18.5–24.9 kg/m^2^)	98	69.0
Overweight (25–29.9 kg/m^2^)	35	24.6
Obese (≥30 kg/m^2^)	9	6.3
Infants (*n* = 104)		
Male	53	51.0
Gestational age at delivery (week), mean ± SD	39.2 ± 1.2	
Full-term birth	103	99.0
Pre-term (<37 week)	1	1.0
Post-term (>42 week)	0	0.0
Delivery method		
Normal delivery	89	85.6
Caesarean section	15	14.4
Birth weight (g), mean ± SD	3188.1 ± 346.2	
Low birth weight	1	1.0
Normal birth weight	103	99.0
Macrosomia	0	0.0
Birth length (cm), mean ± SD	48.7	1.7
Normal (≥48 cm)	81	77.9
Short (<48 cm)	23	22.1

**Table 2:** Infant anthropometrics and body composition (*n* = 104).

Parameter	Value (Mean ± SD)
Age (days)	6.2 ± 1.4
Body weight (g)	3323.3 ± 429.8
Body length (cm)	50.5 ± 2.0
Triceps skin fold (cm)	4.9 ± 1.5
Subscapular skin fold (cm)	5.1 ± 1.8
Thigh skin fold (cm)	5.6 ± 1.6
Fat mass (g)	370.4 ± 168.4
Fat-free mass (g)	2952.9 ± 278.8
Percentage of fat mass (%)	10.7 ± 3.7

**Table 3:** Maternal daily energy, macronutrients, and selected LC-PUFA intake (*n* = 142).

Energy/Nutrients	Mean ± SD/ Median (Q1–Q3)	RDI	Adequate Intake *	
			*n*	%
Energy (kcal/d)	2084.9 ± 800.9	2550 ^1^	56	39.4
Fat (g/d)	84.4 ± 43.9	85 ^1^	100	70.4
Protein (g/d)	70.3 ± 30.6	90 ^1^	51	35.9
Carbohydrate (g/d)	230.5 ± 98.1	380 ^1^	20	14.1
Linoleic acid (g/d)	10.4 (5.5–14.5)	8.5 ^2^	87	61.3
α-linolenic acid (g/d)	0.6 (0.3–0.8)	1.4 ^2^	6	4.2
Eicosapentaenoic acid (mg/d)	2.1 (0.6–10.7)	100 ^2^	2	1.4
Docosahexaenoic acid (mg/d)	38.2 (18.8–67.3)	200 ^2^	4	2.8

RDI—recommended daily intake; SD—standard deviation; ^1^ Indonesian RDI [22]; ^2^ US Department of Agriculture and US Department of Health and Human Services [23]; * adequacy level ≥ 90%.

**Table 4:** Associations of LC-PUFA levels in maternal blood with cord blood and breast milk levels.

	Maternal Blood vs.			
	Cord Blood		Breast Milk	
	r	*p*	r	*p*
Linoleic acid	0.108	0.286	−0.078	0.443
α-linolenic acid	0.069	0.493	0.116	0.255
Eicosapentaenoic acid	0.096	0.341	0.145	0.151
Docosahexaenoic acid	0.011	0.910	−0.001	0.991

r—Spearman correlation coefficient (statistical significance at *p* < 0.05).

**Table 5:** Associations of maternal LC-PUFA intake with levels of LC-PUFAs in maternal and cord blood.

Parameter	Maternal Intake vs.			
	Maternal Blood		Cord Blood	
	r	*p*	r	*p*
Linoleic acid	0.090	0.320	0.426 *	0.000
α-linolenic acid	0.153	0.089	0.314 *	0.001
Eicosapentaenoic acid	0.007	0.938	−0.012	0.905
Docosahexaenoic acid	−0.068	0.448	−0.253 *	0.011

r—Spearman correlation coefficient; * significant correlation (*p* < 0.05).

**Table 6:** Associations of maternal nutrient intake and infant anthropometrics and body composition (*n* = 104).

Maternal Dietary Intake	Infant Anthropometrics and Body Composition					
	Birth Weight	Birth Length	Triceps SFT	Subscapular SFT	Thigh SFT	% Fat Mass
Energy (kcal/d)						
r	0.025	−0.094	0.159	0.128	0.148	0.063
*p*	0.801	0.343	0.107	0.196	0.135	0.527
Protein (g/d)						
r	0.001	−0.011	0.145	0.161	0.169	0.106
*p*	0.990	0.908	0.141	0.102	0.086	0.285
Fat (g/d)						
r	0.077	0.030	0.177	0.194 *	0.157	0.089
*p*	0.438	0.766	0.072	0.048	0.111	0.369
Carbohydrates (g/d)						
r	−0.033	−0.164	0.076	0.006	0.045	−0.023
*p*	0.742	0.097	0.443	0.951	0.653	0.819
Linoleic acid (g/d)						
r	−0.128	0.168	0.203 *	0.203 *	0.254 *	0.099
*p*	0.195	0.088	0.038	0.039	0.009	0.369
α-linolenic acid (g/d)						
r	−0.088	0.185	0.171	0.182	0.213 *	0.081
*p*	0.375	0.059	0.082	0.064	0.030	0.413
Eicosapentaenoic acid (EPA) (mg/d)						
r	−0.156	−0.099	−0.146	−0.118	−0.113	−0.184
*p*	0.113	0.319	0.140	0.233	0.254	0.061
Docosahexaenoic acid (DHA) (mg/d)						
r	−0.221 *	−0.162	−0.142	−0.100	−0.099	−0.192
*p*	0.024	0.099	0.150	0.312	0.320	0.051

SFT—skin fold thickness; * statistically significant correlation (Pearson correlation test, *p* < 0.05); r—Pearson correlation coefficient.

**Table 7:** Associations of maternal blood LC-PUFA levels and infant anthropometrics and body composition (*n* = 102).

Maternal Blood LC-PUFAs	Infant Anthropometrics and Body Composition					
	Birth Weight	Birth Length	Triceps SFT	Subscapular SFT	Thigh SFT	% Fat Mass
LA						
r	−0.026	0.060	−0.036	−0.012	−0.021	−0.037
*p*	0.795	0.550	0.718	0.904	0.835	0.710
ALA						
r	−0.093	−0.060	−0.059	−0.018	0.065	−0.092
*p*	0.350	0.552	0.553	0.861	0.514	0.356
EPA						
r	−0.079	−0.074	0.183	0.189	0.210 *	0.247 *
*p*	0.430	0.461	0.066	0.057	0.034	0.012
DHA						
r	−0.060	−0.164	0.029	0.057	−0.049	−0.078
*p*	0.546	0.099	0.772	0.570	0.625	0.434

SFT—skin fold thickness; LA—linoleic acid; ALA—α-linolenic acid; EPA—eicosapentaenoic acid; DHA—docosahexaenoic acid; * statistically significant correlation (Spearman correlation test, *p* < 0.05); r—Spearman correlation coefficient.

**Table 8:** Associations of cord blood LC-PUFA levels and infant anthropometrics and body composition (*n* = 100).

Cord Blood LC-PUFAs	Infant Anthropometrics and Body Composition					
	Birth Weight	Birth Length	Triceps SFT	Subscapular SFT	Thigh SFT	% Fat Mass
LA						
r	0.002	0.059	0.124	0.109	0.131	0.109
*p*	0.985	0.558	0.221	0.281	0.195	0.279
ALA						
r	−0.007	0.064	0.117	0.058	0.174	0.083
*p*	0.946	0.526	0.245	0.564	0.084	0.412
EPA						
r	−0.074	0.095	−0.134	−0.078	−0.045	−0.081
*p*	0.462	0.349	0.185	0.442	0.658	0.425
DHA						
r	0.272 *	0.024	0.112	0.092	0.114	0.117
*p*	0.006	0.816	0.267	0.364	0.259	0.247

SFT—skin fold thickness; LA—linoleic acid; ALA—α-linolenic acid; EPA—eicosapentaenoic acid; DHA—docosahexaenoic acid; * statistically significant correlation (Spearman correlation test, *p* < 0.05); r—Spearman correlation coefficient.

## Data Availability

Data will be made available upon request to the author Nuri Andarwulan.
